# Nanopore Sequencing in Mycobacterial Diagnostics: Clinical and Laboratory Roles of mNGS and tNGS

**DOI:** 10.3390/diagnostics16121850

**Published:** 2026-06-15

**Authors:** Meng Wang

**Affiliations:** Hangzhou Center for Disease Control and Prevention (Hangzhou Health Supervision Institute), Hangzhou 310021, China; wangm@hzcdc.com.cn; Tel.: +86-135-8880-8301

**Keywords:** nanopore sequencing, mycobacterial diagnostics, tuberculosis, nontuberculous mycobacteria, metagenomic next-generation sequencing, targeted next-generation sequencing, resistance profiling

## Abstract

**Background/Objectives:** Nanopore sequencing is increasingly used in mycobacterial diagnostics, where clinical microbiologists and diagnostic laboratories must decide when broad metagenomic next-generation sequencing (mNGS) or focused targeted next-generation sequencing (tNGS) is most appropriate. This review examined reported clinical and laboratory roles of nanopore mNGS and tNGS in tuberculosis (TB) and nontuberculous mycobacterial (NTM) settings. **Methods:** Targeted searches of PubMed/MEDLINE, Embase, Web of Science Core Collection, and Scopus were refreshed on 4 April 2026. Thirty-five records spanning original clinical studies, evidence syntheses, and guideline-context documents were included. **Results:** Nanopore mNGS is most useful for broad organism detection and diagnostic rescue in unresolved pulmonary and extrapulmonary presentations, particularly when first-line testing is negative, discordant, low-yield, or when mixed infection is suspected. Nanopore tNGS appears better aligned with predefined TB confirmation and resistance-focused workflows because targeted regions allow more standardized interpretation. Agreement is strongest for rifampicin- and isoniazid-related resistance targets. In NTM settings, evidence is stronger for detection and species identification than for disease-level diagnosis. Common implementation constraints include pre-analytical variation, contamination control, host-background interference, inconsistent bioinformatics, and limited workforce capacity. **Conclusions:** A practical tiered approach is supported in which mNGS is positioned mainly for diagnostic rescue and discovery, whereas tNGS is considered for predefined workflows requiring standardized target interrogation and resistance-associated mutation reporting under local validation and quality systems.

## 1. Introduction

Sequencing-based methods now occupy a larger place in mycobacterial diagnosis, especially when conventional microbiology is slow, insufficiently sensitive, or difficult to use for non-sputum and paucibacillary specimens. Within this broader field, nanopore platforms have drawn attention because they offer long reads, near-real-time data generation, and flexible use across research, public-health, and clinical laboratory environments. These features make nanopore systems relevant not only for organism detection but also for resistance-associated target analysis, species-discriminatory sequencing, and clinically interpretable reporting pipelines.

Its role in mycobacterial testing has broadened as well. Initial clinical use centered on metagenomic next-generation sequencing (mNGS) for wide-spectrum pathogen detection, including smear-negative and atypical presentations [1,2,3]. With improved assay design and implementation, targeted next-generation sequencing (tNGS) has become more prominent as a standardized option for rapid TB confirmation and resistance-associated mutation profiling [4,5,6,7,8]. Updated WHO resources for resistance interpretation and diagnostic integration have further reinforced this shift [9,10,11,12]. In addition, a small number of recent illustrative reports published after assembly of the core synthesis set have described nanopore use in smear-negative pulmonary specimens, low-bacillary-burden presentations, and clinically unresolved suspected-TB cohorts, further supporting interest in focused workflow design rather than one-size-fits-all deployment [13,14,15].

From a workflow perspective, mNGS and tNGS serve different purposes. mNGS supports broad, hypothesis-free detection, whereas tNGS concentrates on predefined genomic targets and generally allows more standardized downstream interpretation of resistance-associated and taxonomically informative regions, an orientation that is increasingly aligned with rapid TB-oriented targeted sequencing programs and implementation-focused evaluations [16,17].

This review focuses on nanopore-enabled workflows rather than on NGS categories in general because nanopore platforms combine long reads, near-real-time run monitoring, and deployment flexibility. However, their clinical use still requires attention to evolving chemistry and basecalling pipelines, uneven depth in direct clinical specimens, and variant-calling limits in low-frequency or mixed populations. These constraints are especially important when sequencing outputs are used for resistance reporting, species assignment, or laboratory decision support rather than for detection alone.

This review takes a clinical diagnostics and laboratory medicine perspective on where mNGS and tNGS may currently fit in mycobacterial diagnostics. It examines their reported diagnostic roles, their value for resistance-associated reporting, and the practical problems that arise before, during, and after sequencing. In this manuscript, “diagnostic rescue” refers to sequencing used as an escalation test after first-line microbiological, molecular, or clinical–radiological pathways are negative, discordant, low-yield, or insufficiently explanatory for the patient’s presentation. It does not imply that every conventional test must be negative before sequencing is considered.

Specifically, this review addresses three questions: (i) what diagnostic and resistance-reporting patterns have been described for nanopore mNGS and tNGS in TB/NTM settings; (ii) how do workflow characteristics shape likely clinical use; and (iii) what practical barriers recur across pre-analytical, analytical, and post-analytical phases?

## 2. Methods

This manuscript is an *evidence-informed narrative review* supported by targeted, transparently reported database searches. Quantitative pooling was not performed because study design, specimen type, reference standard, and reported endpoints differed substantially across studies. The review therefore emphasizes clinical and laboratory interpretation rather than pooled effect estimates or definitive comparative-effect claims. Risk-of-bias interpretation of original clinical diagnostic studies was informed by QUADAS-2 domain principles [18].

### 2.1. Review Type and Methodological Boundaries

This review was planned as a narrative synthesis centered on clinical use, laboratory workflow integration, and implementation of nanopore mNGS and targeted workflows in TB/NTM diagnostics. The framing question and broad eligibility domains were specified before database searching. Because specimen types, reference standards, and endpoint definitions varied widely across studies, narrative synthesis was chosen rather than pooled meta-analysis, formal certainty grading, or systematic-review-style effect estimation.

### 2.2. Methodological Transparency and Scope

Because study design, specimen type, reference standard, and endpoint definitions were highly heterogeneous, narrative synthesis was prioritized over pooled quantitative effect estimates. Accordingly, this review reports directional consistency, illustrative metric ranges from selected extractable studies, and diagnostic implementation-relevant constraints.

The search window spanned 1 January 2014 to 4 April 2026, with a final refresh on 4 April 2026. The start year was chosen to capture the early clinical emergence and translational uptake of contemporary nanopore platforms. Eligible records were peer-reviewed indexed publications, including ahead-of-print items with sufficient methodological detail. Preprints, conference abstracts without extractable methods or outcomes, and non-peer-reviewed gray literature were excluded. Full-text synthesis was limited to English-language publications.

Study identification and selection used a single-reviewer workflow with full-dataset verification. One reviewer completed title/abstract and full-text screening, and potentially includable records were rechecked in a second pass against the prespecified criteria. Independent duplicate screening was not undertaken, so inter-reviewer agreement statistics such as Cohen’s kappa were not available. Data extraction followed a predefined framework covering study design, setting, specimen type, sequencing approach, comparator or reference standard, diagnostic and resistance endpoints, turnaround time, and key implementation limitations.

Title/abstract screening was intentionally broad within the prespecified scope domains, whereas full-text assessment confirmed information sufficiency and evidence-layer assignment. The core synthesized evidence set used for results tables, evidence layering, and count-based reporting was fixed after full-text selection and extraction. During revision, a small number of targeted contextual references were added to support discussion of WHO guidance, sequencing implementation, external quality assessment, and cost/resource considerations. These post-review additions were used only for contextual interpretation and were not treated as part of the 35-record core synthesis set. They therefore did not alter the search-flow counts, evidence-layer denominators, Table S1, or the non-pooled diagnostic and resistance-performance summaries.

### 2.3. Search Strategy and Eligibility

PubMed/MEDLINE, Embase, Web of Science Core Collection, and Scopus were searched using three concept groups: (i) nanopore platform terms, (ii) mycobacterial pathogen terms, and (iii) clinical-diagnostic terms. To reduce omission caused by narrow term selection, the diagnostic and workflow block included metagenomic, mNGS, targeted sequencing, tNGS, amplicon sequencing, deep sequencing, long-read sequencing, direct-from-sample sequencing, drug resistance, resistance prediction, susceptibility testing, and related variants. Platform terms included nanopore, Oxford Nanopore, ONT, MinION, GridION, and PromethION. In this review, “tNGS” is used as a workflow-level umbrella label rather than as a claim that all assays were homogeneous. Because studies varied in whether targeted workflows referred to amplicon panels, hybridization/target-enrichment designs, direct-from-specimen assays, or culture-derived sequencing, this definitional variability was treated as an interpretive limitation rather than collapsed into a single assay class.

This review used a multi-type evidence model. Eligible records for synthesis included: (1) original clinical diagnostic studies, (2) systematic reviews/meta-analyses, and (3) high-relevance contextual evidence (narrative reviews, guideline/policy documents, and organizational update notices) used for implementation and interpretation boundaries. Non-mycobacterial studies and non-clinical technical reports without diagnostic outcomes were excluded.

When multiple reports appeared to describe overlapping or closely related study populations, preference was given to the record with the clearest diagnostic endpoint reporting, the most complete methodological detail, or the most directly relevant workflow comparison. Companion reports that remained useful for context were retained narratively but were not allowed to dominate comparative interpretation.

### 2.4. Role of Each Evidence Type in Inference

Original clinical diagnostic studies were treated as the primary evidence base for statements about diagnostic performance, resistance-concordance patterns, and specimen-level workflow feasibility. Systematic reviews/meta-analyses were used to assess whether broad directionality was consistent with the primary-study literature, but they were not pooled with original datasets in the present review. Narrative reviews, guideline/policy documents, and organizational updates were used only to define implementation context, mutation-interpretation boundaries, and reporting considerations; these records were not treated as equivalent to primary diagnostic-accuracy evidence.

### 2.5. Evidence-Type Layering and Missing-Data Handling

To avoid mixing evidence levels, results were stratified into TB-focused tNGS evidence, TB-focused mNGS evidence, and NTM-focused nanopore evidence, with contextual policy/guideline references interpreted separately.

For records with inaccessible full text or non-reporting of numeric endpoints, the record was retained for qualitative context and outcomes were explicitly labeled as [Not extractable]. Documents without primary-cohort diagnostic endpoints were labeled [Not applicable]. These records were excluded from quantitative comparative inference and were not used to make strong comparative claims.

### 2.6. Indirect Comparison Framework

This review did not perform formal cross-platform comparative meta-analysis because reported mNGS and tNGS studies differed substantially in specimen type, patient spectrum, clinical indication, reference standard, and endpoint definition. Accordingly, mNGS–tNGS comparisons were framed qualitatively at workflow level rather than quantitatively at pooled accuracy level. Indirect comparisons were considered only when studies addressed broadly similar clinical tasks, and even then were interpreted as contextual signals of pathway fit rather than as direct comparative performance estimates. For transparency of reporting structure, the search-flow presentation was informed by PRISMA 2020 conventions, without implying that the present manuscript constitutes a fully systematic review; these methodological citations were added to clarify reporting logic rather than to expand the core synthesized study set [19,20].

### 2.7. Risk of Bias Synthesis

Risk-of-bias interpretation used QUADAS-2 domains (patient selection, index test, reference standard, flow/timing), with applicability concerns tracked in parallel. QUADAS-2 signaling logic was tailored to direct-from-specimen sequencing and resistance-concordance endpoints (see the Supplementary Materials, Table S2 caption note). Domain-level patterns are summarized in the main text; study-level judgments are reported in Table S2. Across original clinical studies, recurrent concerns included non-consecutive or incompletely described patient selection, limited index-test protocol transparency, and heterogeneous or imperfect reference standards; these features may overestimate diagnostic accuracy in unselected real-world populations. For evidence layers other than original diagnostic studies (systematic reviews, narrative reviews, guideline/policy documents, and organizational updates), no formal quality-rating tool was applied because these records were used for context and implementation boundaries rather than primary accuracy estimation. To prevent over-interpretation, statements were explicitly framed as data-supported findings versus implementation-oriented inferences.

### 2.8. Literature Search Flow

**Figure 1:**
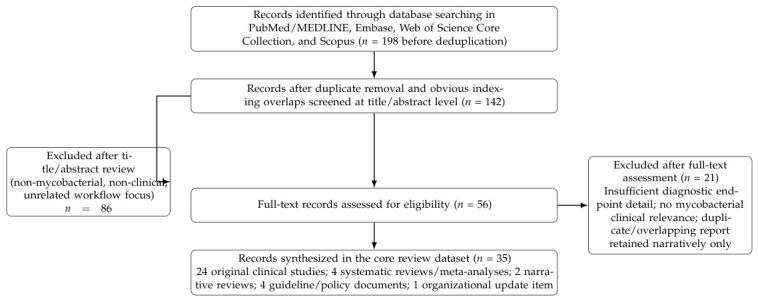
Flow diagram for literature identification and selection.

The literature identification and selection process is summarized in Figure 1.

## 3. Results and Synthesis

### 3.1. Study Portfolio and Evidence Layers

A total of 35 records were synthesized: 24 original clinical studies, 4 systematic reviews/meta-analyses, 2 narrative reviews, 4 guideline/policy documents, and 1 organizational update item. To preserve interpretability, comparative statements about diagnostic performance were primarily grounded in original clinical studies, while non-original records were used to contextualize consistency and implementation direction. The thematic evidence layers shown below are analytic rather than mutually exclusive; therefore, counts across rows should not be summed because a small number of mixed-use records informed more than one layer.

Table 1 summarizes the clinical evidence layers used for synthesis.

Table 2 lists contextual and implementation-oriented evidence used to support interpretation.

### 3.2. From mNGS Discovery to Clinical Triage

Across pulmonary and extrapulmonary TB contexts, mNGS shows clinical value as a broad-range approach when routine pathways are inconclusive. This use case should be interpreted as escalation after negative, discordant, low-yield, or clinically insufficient first-line results rather than as a requirement that all routine tests are uniformly negative. Within the core synthesis set, six primary TB-focused mNGS studies and two supporting meta-analyses contributed to this interpretation. Observational and meta-level evidence indicates meaningful sensitivity gains in selected cohorts (including BALF and smear-negative settings), but cross-study comparability remains constrained by host-background burden, threshold heterogeneity, and pre-analytical variability [38,39,40].

### 3.3. tNGS as the Operational Core for TB Resistance Workflows

Indirect evidence suggests that tNGS workflows may be better suited to predefined TB tasks, especially resistance-oriented pathways. This comparative interpretation is based on workflow fit (target focus, reporting structure, and operational simplicity) rather than direct head-to-head comparisons, and should not be interpreted as proof of universal analytical superiority. Within the 35-record synthesis set, 17 TB-focused tNGS original clinical studies supported diagnostic, resistance-concordance, or direct-from-specimen feasibility statements. Several studies report higher concordance in rifampicin- and isoniazid-associated contexts than in broader multi-drug panels, where depth, locus design, and variant interpretation become more heterogeneous [21,23,31].

### 3.4. Preliminary NTM Detection Evidence and Limits for Disease Diagnosis

NTM-related nanopore evidence remains more limited and less clinically standardized than TB-related evidence. In the core synthesis set, three primary NTM-focused studies and two narrative reviews informed this domain, compared with a larger TB-focused evidence base. Published data suggest potential value for organism detection and species identification, especially in respiratory samples and mixed-infection contexts, but disease-level inference remains substantially weaker than analytical detection or taxonomic assignment [41,45,46,53,54].

### 3.5. NTM Endpoint Boundaries

For NTM, an important distinction must be maintained between sequencing-based organism detection and clinical diagnosis of NTM disease. Colonization, environmental contamination, and uncertain microbiologic significance can all confound interpretation. Accordingly, this review treats NTM findings as stronger for detection/speciation than for direct disease-diagnosis claims and interprets comparative statements in this area conservatively.

### 3.6. Reference-Standard-Stratified Interpretation

Because included studies used different reference standards, findings were interpreted by question type rather than pooled indiscriminately: (i) diagnostic performance versus culture/composite clinical standards, (ii) resistance concordance versus phenotypic DST, and (iii) molecular agreement versus WGS/targeted molecular comparators. This framing reduces over-interpretation across non-equivalent benchmark systems.

### 3.7. Representative Performance Ranges (Narrative, Non-Pooled)

To improve quantitative transparency without formal pooling, illustrative ranges from selected extractable studies are summarized in Table 3. These ranges are intended as orientation aids rather than summary effect estimates.

### 3.8. Evidence-to-Range Mapping Transparency

Table 4 links each representative range to supporting original-study IDs from Table S1. Ranges reflect minimum-to-maximum values across extractable metrics in broadly comparable contexts; records labeled [Not extractable] or [Not applicable] were not used to construct these numeric ranges.

### 3.9. High-Density Evidence Anchor Table

To make claim-to-study linkage more explicit in the main text, Table 5 provides a condensed, high-density anchor map across representative original studies, including specimen context, workflow type (direct versus culture-based), reference standard class, key diagnostic metrics, resistance endpoint reporting, and turnaround-time information where available.

Table 6 summarizes diagnostic role and evidence-strength signals by workflow category.

### 3.10. Direct-from-Specimen Versus Culture-Derived Workflows

Direct-from-specimen approaches are clinically attractive because they may shorten time to actionable information, but their performance depends heavily on bacillary load, host background, extraction method, and contamination control. Culture-derived sequencing can provide cleaner genomic material and broader variant confidence but may delay information return. In practice, the choice between these approaches is likely to depend on whether the clinical priority is immediate triage, resistance support, outbreak resolution, or comprehensive laboratory characterization.

## 4. Implementation Challenges

### 4.1. Pre-Analytical and Laboratory Workflow Issues

Pre-analytical variability remains one of the most important constraints on real-world nanopore performance. Specimen type, storage conditions, decontamination steps, DNA extraction method, and host-background burden all influence downstream yield and interpretability. These problems are magnified in paucibacillary and extrapulmonary specimens, where a technically adequate run may still provide only limited actionable sequence information.

To make these implementation variables more explicit for multicenter planning, Table 7 summarizes technical decision points that should be prespecified when designing or validating nanopore mycobacterial workflows. The table is intended as a planning aid rather than a universal protocol because commercial kits, local extraction systems, specimen transport networks, biosafety requirements, and drug-resistance panels differ across settings.

### 4.2. Interpretation, Reporting, and Governance

Even when sequencing output is technically successful, reporting is not straightforward. Laboratories must decide which organisms or mutations are reportable, how to set thresholds for confidence and contamination exclusion, and how to communicate uncertainty. In TB resistance workflows, this often requires linkage to current WHO mutation catalogues and explicit distinction between validated resistance-associated variants and lower-confidence exploratory calls. For NTM detection, the main reporting risk is over-interpreting organism presence as equivalent to disease.

Bioinformatics inconsistency is not a single problem but a set of workflow-dependent sources of variation. Relevant components include basecalling model and software version, read-length and quality filters, host-read subtraction strategy, reference database content and update frequency, taxonomic classifier choice, alignment and variant-calling parameters, minimum read or depth thresholds, minor-variant cutoffs, negative-control subtraction, and laboratory-specific contamination filters. For tNGS resistance workflows, mutation-catalogue versioning and rules for low-frequency or mixed-population variants are especially important [9,55]. For mNGS workflows, reporting thresholds for low-abundance organisms and environmental mycobacteria require explicit validation because small analytical differences can change whether a result is reported as detected, indeterminate, or likely contaminant.

### 4.3. Access, Cost, and Workforce

Even where sequencing hardware is available, implementation remains constrained by reagent supply chains, bioinformatics support, and trained personnel. This creates a gap between proof-of-concept performance and sustained routine delivery, especially in high-burden settings with constrained resources. Cost discussions should therefore separate capital equipment affordability from recurring consumables, quality-management overhead, and workforce sustainment. For implementation planning, a practical tNGS costing model should at minimum include flow cell use, library/barcoding kit consumption, extraction and amplification reagents, controls and repeat runs, staff time, data-analysis support, and quality-management overhead. Illustrative reagent-only estimates are highly sensitive to multiplexing: when one flow cell and one barcoding kit are shared across a full batch, per-sample sequencing consumable costs may fall substantially, whereas low-throughput or urgent single-sample testing can make the same workflow much more expensive per reportable result. Because public list prices, discounts, import costs, and validation overhead differ by country and institution, this review avoids presenting a single universal cost-effectiveness threshold [56,57].

### 4.4. Reference-Standard Heterogeneity and Interpretive Boundaries

Cross-study comparisons should be interpreted cautiously because reference standards differed substantially across studies (e.g., culture, composite clinical diagnosis, phenotypic DST, molecular assays, and WGS-based comparators). For diagnostic endpoints, culture is an imperfect reference in paucibacillary and extrapulmonary contexts. For resistance endpoints, discordance between phenotypic and genotype-based calls can reflect biological complexity rather than simple test error. For NTM, microbiological detection does not necessarily indicate clinical disease. Accordingly, this review avoids direct cross-context superiority claims and treats many findings as trend-level rather than definitive comparative estimates. These comparator differences are highlighted in the main text and reflected in the risk-of-bias and applicability summary in Table S2.

### 4.5. Platform-Level Versus Workflow-Level Constraints

The practical constraints discussed in this review arise not only from the sequencing platform itself but also from the surrounding workflow, including specimen preparation, assay design, basecalling, alignment strategy, contamination control, mutation interpretation, and reporting pathways. This is one reason why broad platform-level claims are less informative than task-specific workflow comparisons.

## 5. Discussion

The central message from current evidence is not that mNGS and tNGS are competing technologies, but that they represent complementary layers within a clinical diagnostic architecture. mNGS is strongest when pre-test uncertainty is high and pathogen breadth matters; tNGS is strongest when actionability, speed, and standardized resistance interpretation are prioritized. This functional distinction may help explain why tiered workflows are often more plausible than replacement models in current practice.

From a translational perspective, three patterns stand out. First, resistance-focused tNGS is increasingly aligned with routine TB pathways because clinical endpoints and interpretation resources are clearer [9,10]. Second, mNGS is being positioned more selectively as a rescue tool for unresolved or polymicrobial presentations rather than as a universal first-line assay. Third, NTM applications remain less standardized than TB applications, and the lack of uniform disease-level reference standards for NTM is a central reason why detection/speciation findings should not be over-interpreted as diagnostic confirmation of NTM disease.

Several bottlenecks recur across studies: pre-analytical variation, inconsistent bioinformatic filtering, and heterogeneous reporting. These factors affect whether apparently strong analytical performance translates into dependable clinical use, and they help explain why the same assay may have different value across diagnostic ecosystems. Clinical utility should therefore be judged not only by sensitivity and specificity, but also by workflow placement, same-day actionability, reporting consistency, and the laboratory’s ability to sustain quality across specimen types.

A further practical implication is that review conclusions should be interpreted at the level of clinical task rather than sequencing brand alone. Across the included literature, the most decision-relevant contrasts involve broad rescue testing versus predefined targeted testing, direct-from-specimen versus culture-derived workflows, and organism detection versus disease-level inference. Framing the field in this task-oriented way helps reconcile why apparently discordant studies may still support a coherent implementation message: nanopore sequencing is becoming more clinically useful, but its value depends heavily on matching the workflow to the intended diagnostic decision, the specimen context, and the laboratory’s capacity for validation and interpretation.

## 6. Conceptual Implementation Framework (Provisional)

Based on the current evidence base and implementation-oriented guideline context, provisional implementation considerations are outlined rather than formal practice recommendations. A practical selection approach is:

- Choose tNGS when the clinical question is predefined TB confirmation, targeted resistance profiling, or standardized mutation reporting.
- Choose mNGS when the presentation is unresolved, atypical, extrapulmonary with broad differential diagnosis, or polymicrobial infection is suspected.
- Avoid using either approach as a stand-alone disease diagnosis for NTM without clinical, radiologic, and microbiologic correlation.
- Prefer culture-derived sequencing when comprehensive genomic characterization is more important than immediate turnaround; prefer direct-from-specimen workflows when earlier triage is the priority and the specimen has sufficient organism burden.

(1) **Consider a tiered testing pathway.** A practice-oriented interpretation of the current literature is to use mNGS for broad differential diagnosis and difficult cases, while considering tNGS for TB confirmation and resistance profiling in predefined scenarios where indirect evidence suggests better pathway fit. Evidence basis: indirect comparative interpretation, stronger for TB resistance than for NTM diagnosis.
(2) **Prioritize pre-analytical standardization.** Laboratories should define and audit protocols for specimen acceptance, host-background mitigation, contamination prevention, and minimum sequencing quality criteria. Evidence basis: recurrent cross-study implementation bottlenecks.
(3) **Harmonize interpretation and reporting where feasible.** Resistance calls should be mapped to current WHO catalogues, and reports should clearly distinguish high-confidence resistance-associated variants from exploratory findings [9,47]. Evidence basis: moderate for selected TB drug classes, limited for broader panels and NTM disease endpoints.
(4) **Build multidisciplinary governance where resources permit.** Sustainable implementation requires coordinated oversight by clinicians, microbiologists, molecular diagnosticians, and bioinformaticians, with periodic external quality assessment. Evidence basis: implementation logic and quality-systems requirements rather than head-to-head effectiveness trials.
(5) **Prioritize equity-focused deployment research.** Future multicenter work should include implementation-effectiveness and cost analyses in high-burden, resource-constrained settings. Evidence basis: currently limited direct economic and outcome-trial data.

## 7. Limitations of This Review

This review has several limitations that should frame interpretation of its conclusions. First, screening and full-text assessment were performed by a single reviewer with verification rather than by independent dual reviewers; therefore, missed records and subjective selection effects cannot be excluded, and no inter-reviewer agreement statistic such as Cohen’s kappa was available. Second, the included literature was highly heterogeneous in study design, specimen type, clinical indication, reference standard, endpoint definition, and use of the tNGS label across different targeted assay designs. Although evidence-layer stratification and cautious indirect comparison were used to reduce over-interpretation, residual heterogeneity limits direct comparability across studies and precludes strong pooled or superiority-style inferences.

Third, this review intentionally integrates multiple evidence types, including original clinical studies, evidence syntheses, and policy-context documents. To reduce mixing of evidence levels, comparative statements were anchored primarily in original clinical datasets, whereas non-original sources were used to define consistency and implementation boundaries. Even so, several records lacked extractable numerical endpoints and therefore informed qualitative context only. Publication bias toward favorable diagnostic performance is also possible in this rapidly evolving field, and the English-language full-text restriction may have reduced capture of relevant non-English studies.

Finally, the source literature itself was often incompletely reported. The predominance of “unclear” QUADAS-2 judgments, particularly for patient selection, index-test conduct, and flow/timing, indicates that some apparent performance signals should be interpreted cautiously. Accordingly, the present review is better suited to clarifying likely clinical roles and implementation boundaries than to serving as a substitute for a fully systematic comparative review or formal guideline-development process.

## 8. Conceptual Workflow Model

**Figure 2:**
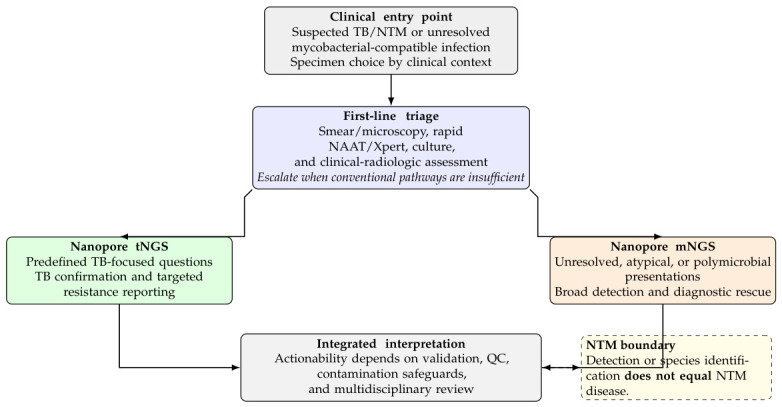
Proposed clinical positioning of nanopore mNGS and tNGS in mycobacterial diagnostic pathways. Sequencing is framed as an escalation step after first-line triage, with distinct roles for targeted and broad workflows.

**Figure 3:**
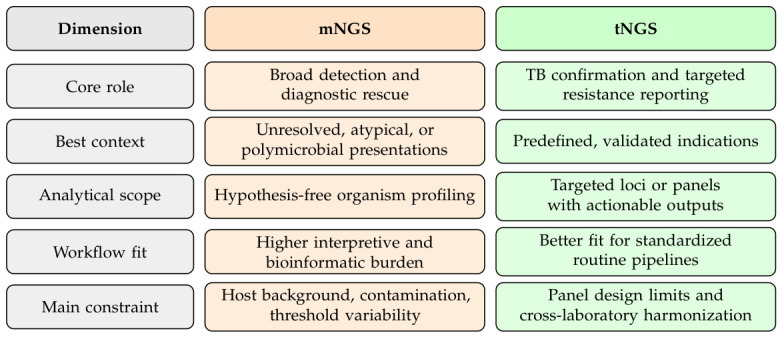
Practical comparison framework for mNGS and tNGS in mycobacterial diagnostics. The figure emphasizes differentiated use scenarios rather than head-to-head superiority.

Based on the evidence reviewed here, a practical tiered model is more defensible than a single universal sequencing pathway. First-line molecular and culture-based methods remain the initial triage step. Within that framework, nanopore tNGS is best aligned with predefined TB-focused questions such as confirmation and targeted resistance reporting, whereas nanopore mNGS is better reserved for unresolved, atypical, or polymicrobial presentations requiring broader organism detection. Figure 2 summarizes this proposed clinical positioning in a simplified practice-oriented framework, and Figure 3 highlights the main trade-offs between the two sequencing strategies.

Figure 3 complements Figure 2 by summarizing the practical trade-offs between broad and targeted nanopore workflows across the main decision dimensions used in this review.

## 9. Clinical Pathway Implications

A practical implication of the current evidence base is that sequencing should remain embedded within the broader diagnostic pathway rather than treated as a stand-alone replacement strategy. In simplified terms, tNGS fits narrower validated TB-focused questions, whereas mNGS fits broader rescue-oriented questions after conventional testing is insufficient. As shown in Figure 3, these roles differ in analytical scope, workflow fit, interpretive burden, and dominant implementation constraints. For diagnostic laboratories, this implies different validation, contamination-control, reporting, and staffing requirements rather than simple platform substitution.

This perspective also clarifies why direct comparisons between mNGS and tNGS are difficult to interpret: the two approaches often occupy different clinical roles. In extrapulmonary TB and NTM-related presentations, where specimen scarcity, low bacillary burden, and uncertain disease attribution complicate interpretation, the intended decision point should be stated explicitly—diagnostic rescue, species identification, resistance support, or broader infection profiling. Doing so would improve study comparability and reduce over-interpretation of organism detection as clinically meaningful disease.

## 10. Future Research Priorities

Five research priorities stand out. First, multicenter head-to-head studies with standardized specimen processing and unified reference standards are needed to improve comparability across cohorts. Such evidence would make any preference for tNGS in standardized TB workflows more definitive if prospective studies showed reproducible superiority or non-inferior diagnostic yield with faster actionable resistance reporting, lower indeterminate-call rates, improved treatment modification or time-to-effective-therapy outcomes, and acceptable cost-effectiveness across independent laboratories. Second, implementation-focused trials should assess clinical impact measures such as time to effective therapy, treatment modification, and patient outcomes rather than relying only on analytical metrics. Third, economic analyses are needed to clarify cost-effectiveness thresholds in both high-resource and high-burden settings. Fourth, external quality assessment programs should be strengthened for both wet-lab and bioinformatics components of nanopore workflows. Current EQA infrastructure is better established for conventional mycobacterial culture, molecular TB assays, and some sequencing-based surveillance or WGS activities than for direct clinical nanopore mycobacterial mNGS/tNGS workflows, so laboratories adopting these assays may need interim local proficiency testing, shared contrived panels, negative-control exchange, and bioinformatics challenge datasets until dedicated schemes mature [51,55]. Fifth, consensus reporting frameworks are needed for low-frequency variants, mixed infections, and uncertain resistance calls so that interpretation does not drift across laboratories [16,17,58]. Additional recent reports and WHO contextual resources outside the core synthesized set support the need for clearer assay-selection logic and implementation standards as tNGS moves toward routine diagnostic integration [11,12,14]. Broader reviews were used only for contextual framing and not as primary diagnostic evidence.

A related priority is more task-specific study design. Future work should distinguish pathogen detection, species identification, resistance prediction, and disease-level clinical diagnosis rather than combining these endpoints into a single performance measure. This distinction is especially important in NTM settings, where analytical detection of an organism is not the same as establishing clinical disease. Clearer endpoint definitions would improve evidence synthesis and help laboratories align assay design with the clinical decision the test is meant to support.

For NTM specifically, future studies should be prospective, multicenter, and designed around clinically adjudicated disease definitions rather than detection alone. Useful designs would enroll consecutive patients with suspected NTM pulmonary or extrapulmonary disease, apply standardized specimen handling and culture/molecular comparators, include negative and non-NTM respiratory-disease controls, and prespecify how sequencing results are integrated with clinical, radiologic, and microbiologic criteria. Sample-size planning should be endpoint-driven: species-identification studies require adequate representation of common and clinically important rare species, whereas disease-diagnosis studies require enough confirmed NTM disease and non-disease comparator cases to estimate specificity and positive predictive value with acceptable precision. Studies should also report contamination controls, database versions, read thresholds, indeterminate-call rules, and the proportion of cases in which sequencing changed management.

## 11. Conclusions

Nanopore approaches in mycobacterial diagnostics are moving beyond proof-of-concept and toward broader real-world evaluation. Current evidence suggests that mNGS is most helpful for broad diagnostic rescue and complex differential diagnosis, whereas tNGS may offer better operational fit in predefined settings that require TB confirmation, standardized target interrogation, and earlier resistance-informed decisions. In NTM settings, the evidence is stronger for detection and species identification than for disease-level diagnosis, so organism detection should not be treated as equivalent to NTM disease. Because most comparisons between mNGS and tNGS remain indirect and heterogeneous, any preference for tNGS in standardized workflows should be regarded as provisional until prospective head-to-head studies demonstrate reproducible clinical utility, resistance-reporting actionability, and implementation value across independent laboratories. For clinical laboratories, the strongest current use case is pathway design, validation planning, and quality-system development rather than immediate universal replacement of existing methods. Further progress will depend on better workflow standardization, bioinformatic interpretation, and implementation-focused studies.

## Figures and Tables

**Table 1:** Clinical evidence layers used for synthesis.

Evidence Layer	Evidence Tier	Records (*n*)	Typical Specimen	Typical Comparator	Main Outputs Used in Synthesis
TB-focused tNGS	Primary clinical evidence	17	Sputum, BALF, tissue, mixed non-sputum	Culture, phenotypic DST, Xpert, molecular tests	Diagnostic Se/Sp patterns, resistance concordance, and direct-from-specimen feasibility across representative contexts [21,22,23,24,25,26,27,28,29,30,31,32,33,34,35,36,37]
TB-focused mNGS	Primary + synthesis support	6 primary studies (+2 meta-analyses)	BALF, extrapulmonary samples	Culture, composite diagnosis, Xpert	Rescue-diagnostic role and sensitivity signals in selected low-yield pulmonary and extrapulmonary cohorts [38,39,40,41,42,43,44]
NTM-focused nanopore evidence	Preliminary primary evidence	3 primary studies (+2 narrative reviews)	Respiratory samples	Culture and molecular methods	Species identification, mixed-infection detection, and cautious disease-level interpretation [41,45,46]

**Table 2:** Contextual evidence layers used for implementation and interpretation boundaries.

Evidence Layer	Evidence Tier	Records (*n*)	Typical Specimen	Typical Comparator	Main Outputs Used in Synthesis
Guideline/policy context (WHO and related)	Background implementation context	4 core records (+additional WHO contextual documents cited outside the core set)	Not specimen-based	Not a diagnostic comparator framework	Implementation boundaries, mutation interpretation, target-product expectations, and reporting harmonization [47,48,49,50,51,52]
Organizational update context	Background implementation context	1	Not specimen-based	Not a diagnostic comparator framework	Contextual implementation signals; not weighted as guideline evidence

**Table 3:** Representative non-pooled performance ranges by domain.

Domain	Context	Representative Range	Note
TB diagnosis (tNGS)	Respiratory/non-sputum cohorts	Sensitivity 83–93%; specificity 84–99%	Reflects heterogeneous cohorts and references
TB diagnosis (mNGS)	BALF/EPTB-enriched cohorts	Sensitivity 56–79%; specificity often high	Strongly context-dependent
TB resistance (tNGS)	Drug-class concordance	Selected overall/key-mutation agreement 94–100%; drug-class accuracy reported as 43–93% in extractable studies	Not pooled; strongest for RIF/INH, assay- and panel-dependent
NTM nanopore studies	Identification-focused settings	No robust representative range	Detection/identification are not equivalent to NTM disease diagnosis

**Table 4:** Mapping of illustrative ranges to supporting original studies.

Domain Statement in Table 3	Supporting Original-Study IDs (Table S1)	Construction Rule	Comparability Judgment
TB diagnosis (tNGS): Se 83–93%, Sp 84–99%	6, 10, 15, 16, 17, 23	Extractable Se/Sp values only	Moderate within-domain comparability; cross-study heterogeneity remains substantial
TB diagnosis (mNGS): Se 56–79%; specificity often high	27, 28, 29, 31	TB-focused cohorts; composite-only endpoints retained narratively	Limited comparability because of enriched case mix and differing references
TB resistance (tNGS): selected agreement 94–100%; drug-class accuracy 43–93%	9, 13, 22	Extractable resistance agreement or accuracy values only; no pooled weighting	Moderate within-assay comparability; limited across assays and drug classes

**Table 5:** High-density evidence anchor table for representative original studies (selected to represent major evidence domains and workflow contrasts based on extractable diagnostic/resistance/TAT information; not an exhaustive list of all included original studies).

Study ID	Workflow	Specimen Context	Reference Standard Class	Key Diagnostic Signal	Resistance Endpoint	TAT/Operational Note
6	tNGS	Native sputum	Phenotypic DST/clinical lab workflow	High resistance-prediction utility in field implementation	Multi-drug panel concordance	Native-sputum field use
10	tNGS	Mixed clinical specimens	Molecular/culture comparator	Broad direct-from-specimen feasibility	Mutation detection	Side-by-side platform comparison
15	tNGS	Non-sputum	Composite diagnostic comparator	High TB detection utility in non-sputum cohorts	Resistance support	Multicenter prospective design
16	tNGS	Mixed multicenter	Culture/DST	Strong TB detection and resistance signal	Multi-drug panel	Retrospective multicenter
17	tNGS	Smear-negative pulmonary	Clinical/culture composite	Useful in smear-negative PTB	Limited concordance detail	Prospective design
23	tNGS	BALF sputum-scarce PTB	Culture/clinical comparator	Good BALF diagnostic utility	Not primary endpoint	BALF-focused population
27	mNGS	BALF PTB	Culture/composite	Useful rescue role in pulmonary TB	Not primary endpoint	BALF-focused
28	mNGS	Pulmonary TB	Composite diagnostic comparator	Meta-level sensitivity signal	Not primary endpoint	Review-level synthesis anchor
29	mNGS	Pulmonary BALF	Culture/composite	Positive rescue utility	Not primary endpoint	Retrospective BALF analysis
31	mNGS	PTB/mixed contexts	Meta-analysis	Broad supportive sensitivity direction	Not primary endpoint	Meta-level synthesis anchor

**Table 6:** Diagnostic and resistance endpoint signals for the representative studies in Table 5.

Study ID	Main Endpoint Class	Primary Signal Direction	Interpretation Note
6	Resistance concordance	Favorable	Best interpreted for implementation-oriented DST support rather than universal diagnostic replacement
10	Direct diagnostic + resistance	Favorable	Supports feasibility; not a single-platform superiority claim
15	TB diagnosis (non-sputum)	Favorable	Non-sputum context is clinically important but heterogeneous
16	TB diagnosis + resistance	Favorable	Supports expanded tNGS role in predefined workflows
17	Smear-negative PTB diagnosis	Favorable	Particularly relevant to low-yield pulmonary triage
23	BALF diagnosis	Favorable	BALF findings should not be generalized to all respiratory settings
27	Rescue-diagnostic mNGS use	Mixed favorable	Best interpreted as escalation-step evidence
28	Meta-level pulmonary mNGS synthesis	Mixed favorable	Heterogeneity limits direct pooling interpretation in this review
29	BALF mNGS diagnosis	Favorable	Enriched pulmonary cohort; rescue context remains important
31	Meta-level mNGS synthesis	Mixed favorable	Supports directional signal, not pooled quantitative equivalence

**Table 7:** Technical implementation variables relevant to multicenter nanopore mycobacterial diagnostic studies.

Domain	What Should Be Prespecified	Main Challenge	Practical Planning Implication
DNA extraction	Decontamination, lysis method, bead-beating or enzymatic steps, manual versus automated extraction, extraction controls, and kit/reagent costs	Mycobacterial cell-wall disruption and host background vary by specimen	Compare yield, inhibition, hands-on time, biosafety needs, and per-sample reagent cost before multicenter rollout
Specimen collection/transport	Respiratory versus extrapulmonary specimen type, transport medium, cold-chain conditions, time to processing, and rejection criteria	WHO-endorsed tNGS use is clearest for respiratory TB specimens, whereas extrapulmonary specimens are often low-volume and paucibacillary	Use first-line smear, NAAT/Xpert, culture, and clinical assessment before sequencing; define escalation rules separately for respiratory and extrapulmonary samples
Bacillary load assessment	Smear grade, Xpert/NAAT cycle threshold where available, culture positivity, or mycobacterial qPCR	Low bacillary burden increases failed or indeterminate sequencing and reduces resistance-call confidence	qPCR/NAAT Ct is preferable for quantitative triage where available; smear remains useful for rapid low-resource stratification
Target design and drugs	Commercial kit versus custom primers/probes, included loci, mutation catalogue version, and drug panel	Fixed panels may lag behind newer drugs or emerging resistance mechanisms; custom panels require stronger validation	Define target drugs prospectively and update panels against WHO catalogues and local epidemiology; report uncovered drugs explicitly
Phenotypic DST comparator	Which drugs receive phenotypic DST, including newer drugs such as bedaquiline and pretomanid where feasible	Not all studies or settings perform phenotypic DST for all drugs, and newer-drug DST capacity is uneven	Conceptual workflows should distinguish validated resistance calls from drugs without adequate phenotypic or catalogue support
Batching and throughput	Number of samples per flow cell, barcode strategy, urgent single-sample pathway, and repeat-run rules	High multiplexing lowers reagent cost but may reduce depth per sample and delay urgent reporting	Prespecify minimum reads/depth per sample and a reflex strategy for low-yield barcodes
Run and extraction QC	DNA quantity/quality, negative and positive controls, internal amplification controls, sequencing yield, read quality, barcode balance, and contamination review	A run can be technically successful but clinically uninterpretable for a low-burden sample	Report sample-level success, indeterminate, and repeat rates rather than only run-level success
Bioinformatics QC	Basecalling version, read filters, database version, alignment/variant caller, depth thresholds, minor-variant cutoffs, and contamination filters	Pipeline changes can change organism or resistance calls	Lock and version pipelines during validation; revalidate after major software, database, or chemistry updates
Platform choice	MinION, GridION, or PromethION according to throughput, infrastructure, and turnaround needs	Platform choice affects batching, staffing, maintenance, and cost structure more than the conceptual clinical role alone	MinION-type workflows fit flexible low-throughput deployment; GridION/PromethION may fit higher-throughput centralized laboratories
Lineage and epidemiology	Whether lineage, mixed infection, or transmission-related outputs are required	Lineage may contextualize resistance and epidemiology but is not always needed for immediate clinical reporting	Separate clinical resistance reporting from optional lineage/surveillance outputs and validate each reporting layer
Depth and coverage	Minimum depth per target, breadth of coverage, uniformity, and sample-type-specific success thresholds	Paucibacillary and extrapulmonary samples often have lower and less uniform coverage	Report coverage by specimen type and avoid resistance calls where target depth is below validated thresholds
EQA/proficiency testing	Sample-based panels, culture-derived DNA, contrived materials, negative controls, and bioinformatics challenge datasets	Concordance can be defined at different levels: detection, coverage, run success, variant calling, or resistance prediction	Use staged EQA: wet-lab sample processing where possible plus separate bioinformatics and resistance-interpretation challenges
Clinical interpretation and cost	Report format, uncertainty language, clinician-facing actionability, and cost components including reagents, flow cells, staff, repeats, and informatics	Cost-effectiveness depends on batching, local prices, and whether results change treatment decisions	Link reporting to actionable clinical questions and track treatment modification, turnaround time, and failed-run costs
Failed or indeterminate runs	Criteria for repeat extraction, repeat sequencing, alternative testing, and final reporting language	Failures may reflect extraction inhibition, low organism burden, contamination, or insufficient depth	Predefine reflex pathways to culture, NAAT, phenotypic DST, or repeat sequencing; report failure rates transparently

## Data Availability

This review did not generate new primary datasets. Extracted study-level characteristics, search materials, and methodological summaries are presented in the manuscript and Supplementary Materials, including Table S1, and will be available with the submitted article materials.

## Supplementary Materials

The following supporting information can be downloaded at https://www.mdpi.com/article/10.3390/diagnostics16121850/s1. Table S1: study-level characteristics for retained records. Table S2: QUADAS-2 domain summary for original clinical diagnostic studies. File S1: Database search strategies for PubMed/MEDLINE, Embase, Web of Science Core Collection, and Scopus. File S2: Predefined extraction framework. File S3: screening disposition summary with exclusion-category details. For readability, these materials are not reproduced in full in the main manuscript. They are referenced in the Methods and Results sections as supporting documentation for this evidence-informed narrative review rather than as a substitute for a formally conducted systematic review.
