# Supplementary material for: Nanopore Sequencing in Mycobacterial Diagnostics: Clinical and Laboratory Roles of mNGS and tNGS
Source: Diagnostics (Basel). 2026 Jun 15;16(12):1850. doi: 10.3390/diagnostics16121850 (PMC13297816; doi:10.3390/diagnostics16121850)
Supplement: Supplementary file 1 [file diagnostics-16-01850-s001.zip › diagnostics-4290692-supplementary/File_S1_Search_Strategies.pdf]

File S1: Database Search Strategies

These searches were developed to support a structured narrative review of nanopore-enabled workflows in mycobacterial diagnostics. The search logic combined three concept blocks: (i) nanopore platform terms, (ii) mycobacterial pathogen terms, and (iii) clinical-diagnostic or sequencing-workflow terms. Searches were run across PubMed/MEDLINE, Embase, Web of Science Core Collection, and Scopus. The search window covered 1 January 2014 to 4 April 2026, with a final search refresh on 4 April 2026. Terms were adapted to the syntax of each database.

Core Search Concept Blocks

Platform block: nanopore; Oxford Nanopore; ONT; MinION; GridION; PromethION.

Pathogen block: mycobacter*; *Mycobacterium tuberculosis*; tuberculosis; TB; nontuberculous mycobacteria; non-tuberculous mycobacteria; NTM.

Diagnostic/workflow block: diagnosis; diagnostic; detection; clinical; metagenomic; metagenomic next-generation sequencing; mNGS; targeted sequencing; targeted next-generation sequencing; tNGS; amplicon sequencing; deep sequencing; long-read sequencing; direct-from-sample sequencing; drug resistance; resistance prediction; susceptibility testing.

Representative PubMed/MEDLINE Search

```
(
  nanopore OR "Oxford Nanopore" OR ONT OR MinION OR GridION OR PromethION
)
AND
(
  mycobacter* OR "Mycobacterium tuberculosis" OR tuberculosis OR TB
  OR "nontuberculous mycobacteria" OR "non-tuberculous mycobacteria" OR NTM
)
AND
(
  diagnosis OR diagnostic OR detection OR clinical OR metagenomic
  OR "metagenomic next-generation sequencing" OR mNGS
  OR "targeted sequencing" OR "targeted next-generation sequencing" OR tNGS
  OR "amplicon sequencing" OR "deep sequencing" OR "long-read sequencing"
  OR "direct-from-sample sequencing" OR "drug resistance"
  OR "resistance prediction" OR "susceptibility testing"
)
AND ("2014/01/01"[Date - Publication] : "2026/04/04"[Date - Publication])
```

Representative Embase Search Logic

```
('nanopore' OR 'oxford nanopore' OR ont OR minion OR gridion OR promethion)
```

```
AND
(mycobacter* OR 'mycobacterium tuberculosis' OR tuberculosis OR tb
 OR 'nontuberculous mycobacteria' OR 'non-tuberculous mycobacteria' OR ntm)
AND
(diagnos* OR detect* OR clinical OR metagenomic OR 'metagenomic next generation sequencing'
 OR mngs OR 'targeted sequencing' OR 'targeted next generation sequencing' OR tngs
 OR 'amplicon sequencing' OR 'deep sequencing' OR 'long-read sequencing'
 OR 'direct-from-sample sequencing' OR 'drug resistance' OR 'resistance prediction'
 OR 'susceptibility testing')
AND [2014-2026]/py
```

Representative Web of Science Core Collection Search Logic

```
TS=(nanopore OR "Oxford Nanopore" OR ONT OR MinION OR GridION OR PromethION)
AND TS=(mycobacter* OR "Mycobacterium tuberculosis" OR tuberculosis OR TB
        OR "nontuberculous mycobacteria" OR "non-tuberculous mycobacteria" OR NTM)
AND TS=(diagnos* OR detect* OR clinical OR metagenomic OR mNGS
        OR "targeted next-generation sequencing" OR tNGS
        OR "long-read sequencing" OR "drug resistance"
        OR "resistance prediction" OR "susceptibility testing")
Refined by publication years 2014--2026.
```

Representative Scopus Search Logic

```
TITLE-ABS-KEY(
  nanopore OR "Oxford Nanopore" OR ONT OR MinION OR GridION OR PromethION
)
AND TITLE-ABS-KEY(
  mycobacter* OR "Mycobacterium tuberculosis" OR tuberculosis OR TB
  OR "nontuberculous mycobacteria" OR "non-tuberculous mycobacteria" OR NTM
)
AND TITLE-ABS-KEY(
  diagnos* OR detect* OR clinical OR metagenomic OR mNGS
  OR "targeted sequencing" OR "targeted next-generation sequencing" OR tNGS
  OR "amplicon sequencing" OR "long-read sequencing"
  OR "drug resistance" OR "resistance prediction" OR "susceptibility testing"
)
AND PUBYEAR > 2013
```

Additional Notes

Searches were supplemented by manual review of reference lists and citation tracking for high-relevance records. Terminology was intentionally broader at title/abstract screening stage to reduce omission of clinically relevant nanopore studies using heterogeneous assay labels.
