# Supplementary material for: Nanopore Sequencing in Mycobacterial Diagnostics: Clinical and Laboratory Roles of mNGS and tNGS
Source: Diagnostics (Basel). 2026 Jun 15;16(12):1850. doi: 10.3390/diagnostics16121850 (PMC13297816; doi:10.3390/diagnostics16121850)
Supplement: Supplementary file 1 [file diagnostics-16-01850-s001.zip › diagnostics-4290692-supplementary/File_S2_Extraction_Framework.pdf]

File S2: Predefined Extraction Framework

The following variables were prespecified for structured extraction from retained records. Not all variables were applicable to every evidence type.

Domain	Variables captured
Study identification	First author, publication year, citation key, country or setting, journal, evidence type.
Study design	Prospective or retrospective design, single-center or multicenter setting, clinical versus contextual evidence layer.
Population and specimen context	Suspected TB, confirmed TB, extrapulmonary TB, pulmonary infection cohort, NTM-focused cohort, specimen type, paucibacillary or smear-negative context where reported.
Sequencing workflow	Nanopore platform, mNGS versus targeted workflow, direct-from-sample versus cultured material, broad workflow description, reported turnaround-time information.
Comparator or reference context	Culture, Xpert or other molecular assays, composite clinical reference, phenotypic DST, comparator sequencing platform, or contextual policy benchmark.
Diagnostic endpoints	Sensitivity, specificity, positive/negative predictive values, agreement, AUC, positivity rate, species identification performance, or qualitative diagnostic signal.
Resistance-related endpoints	Reported resistance-associated targets, mutation concordance, rifampicin or isoniazid-related results, broader resistance-prediction statements, and major interpretation caveats.
Implementation issues	Specimen adequacy, contamination risk, host-background interference, library-preparation constraints, bioinformatic variability, workforce or laboratory-capacity issues.
Evidence-use note	Whether the record contributed to primary diagnostic interpretation, contextual interpretation only, or implementation-boundary discussion.
Extractability tag	Extractable numeric endpoint, `[Not extractable]`, or `[Not applicable]` as defined in the main manuscript.

Extraction Approach

Extraction was performed by a single reviewer using this prespecified framework and was aligned with the evidence-layering strategy described in the main manuscript. When records lacked primary-cohort diagnostic endpoints or sufficient numerical detail, they were retained for qualitative context only and were not used to support strong comparative claims.
