# Supplementary material for: Nanopore Sequencing in Mycobacterial Diagnostics: Clinical and Laboratory Roles of mNGS and tNGS
Source: Diagnostics (Basel). 2026 Jun 15;16(12):1850. doi: 10.3390/diagnostics16121850 (PMC13297816; doi:10.3390/diagnostics16121850)
Supplement: Supplementary file 1 [file diagnostics-16-01850-s001.zip › diagnostics-4290692-supplementary/File_S3_Screening_Disposition.pdf]

File S3: Screening Disposition Summary

This summary is provided to document study identification and selection for an evidence-informed narrative review supported by transparent database searches. It is intended as transparency documentation rather than as a full formal systematic-review flow package.

Identification and Screening Totals

- Records identified from database searching (PubMed/MEDLINE, Embase, Web of Science Core Collection, and Scopus): $n = 198$ before deduplication.
- Records remaining after duplicate removal and obvious indexing-overlap handling: $n = 142$.
- Records excluded after title/abstract screening: $n = 86$.
- Full-text articles assessed for eligibility: $n = 56$.
- Full-text articles excluded: $n = 21$.
- Records retained for final synthesis: $n = 35$.

Evidence Composition of Retained Records

- Original clinical studies: $n = 24$.
- Systematic reviews/meta-analyses: $n = 4$.
- Narrative reviews: $n = 2$.
- Guideline or policy documents: $n = 4$.
- Organizational update item: $n = 1$.

Main Exclusion Categories During Screening

At title/abstract and full-text review, records were commonly excluded for one or more of the following reasons:

- non-mycobacterial focus;
- non-clinical technical report without diagnostic endpoint relevance;
- non-nanopore sequencing focus;
- conference abstract, preprint, or brief item without extractable methodological detail;
- duplicate or substantially overlapping report with less complete diagnostic reporting;
- insufficient full-text information for evidence-layer assignment or endpoint interpretation.

Screening Notes

Title and abstract screening was intentionally broad within the prespecified scope domains. Full-text assessment was used to confirm information sufficiency, evidence-layer assignment, and whether retained records could contribute to diagnostic interpretation, implementation context, or both. Screening and verification were performed in a single-reviewer workflow with a second verification pass, as described in the main manuscript.
