# Supplementary material for: Nanopore Sequencing in Mycobacterial Diagnostics: Clinical and Laboratory Roles of mNGS and tNGS
Source: Diagnostics (Basel). 2026 Jun 15;16(12):1850. doi: 10.3390/diagnostics16121850 (PMC13297816; doi:10.3390/diagnostics16121850)
Supplement: Supplementary file 1 [file diagnostics-16-01850-s001.zip › diagnostics-4290692-supplementary/Table_S1_Study_Matrix.pdf]

Table S1: Study-level characteristics of 35 included records (fields marked NR indicate unavailable details; "Mixed/Not clearly specified" denotes insufficient gr anularity in so urce reports).

ID	Author, year	Design	Specimen	Seq	Main finding / endpoint note
1	Schwab et al., 2025	Observational diagnostic study	Sputum	tNGS	Field evaluation of nanopore targeted next-generation sequencing to predict drug-resi...; [Not extractable] Se/Sp/accuracy numerical values unavailable from currently accessible source (full text required)
2	Murthy et al., 2025	Narrative review/scoping review	Mixed/Not clearly specified	Nanopore sequencing (unspecified)	Diagnosis of nontuberculous mycobacterial infections using genomics and artificial in...; Review article (no primary diagnostic accuracy cohort)
3	Zhao et al., 2025	Observational diagnostic study	Mixed/Not clearly specified	Nanopore sequencing (unspecified)	The diagnostic value of third-generation nanopore sequencing in non-tuberculous mycob...; Se/Sp/PPV/NPV/AUC/kappa = 81.3%/98.8%/99.1%/76.6%/0.901/0.759
4	Maleki et al., 2025	Narrative review	Mixed/Not clearly specified	Nanopore sequencing (unspecified)	The growing impact of nontuberculous mycobacteria: {A} multidisciplinary review of ec...; Review article (no primary diagnostic accuracy cohort)
5	Murphy et al., 2023	Observational diagnostic study	Mixed/Not clearly specified	tNGS	Direct detection of drug-resistant mycobacterium tuberculosis using targeted next gen...; N=72 primary specimens (55 cultures); [Not extractable] Se/Sp/accuracy numerical values unavailable from currently accessible source; TAT: two to three days
6	Yang et al., 2026	Prospective observational study	Non-sputum specimens (BALF-dominant)	tNGS	Accuracy of nanopore-based targeted next-generation sequencing assay for detection of...; MRS (microbiological reference standard)-based Se/Sp = 93.4%/93.2% (N=701)
7	Carandang et al., 2025	Systematic review/meta-analysis	Mixed/Not clearly specified	tNGS	Diagnostic accuracy of nanopore sequencing for detecting mycobacterium tuberculosis a...; Pooled Se/Sp/PPV/NPV/AUC = 88.61%/93.18%/94.71%/84.33%/0.932
8	Gui et al., 2026	Observational diagnostic study	Mixed/Not clearly specified	tNGS	Performance evaluation of targeted nanopore sequencing in non-tuberculous mycobacteri...; 18/50 full concordance; F1 tNGS/mNGS/Sanger = 0.927/0.896/0.543
9	Yu et al., 2025	Retrospective observational study	Mixed/Not clearly specified	tNGS	Nanopore sequencing for precise detection of \{}textit{mycobacterium tuberculosis} and ...; DR accuracy 42.9–93.0%; key sensitivities reported (RIF/INH/EMB/FQ/STR)
10	Yan et al., 2024	Prospective observational study	Respiratory specimens	Nanopore sequencing (unspecified)	Nanopore sequencing for smear-negative pulmonary tuberculosis-a multicentre prospecti...; Se/Sp/Acc/NPV/AUC = 83.33%/84.62%/83.78%/73.33%/0.840
11	Ren et al., 2024	Observational diagnostic study	Mixed/Not clearly specified	tNGS	Potential of nanopore sequencing for tuberculosis diagnosis and drug resistance detec...; Sensitivity 0.786, AUC 0.867, kappa 0.488; outperformed qPCR/RNA/culture/smear
12	Hall et al., 2023	Observational diagnostic study	Mixed/Not clearly specified	Nanopore sequencing (unspecified)	Evaluation of nanopore sequencing for mycobacterium tuberculosis drug susceptibility ...; [Not extractable] Diagnostic performance described qualitatively; numerical metrics unavailable from currently accessible source
13	Cabibbe et al., 2024	Observational diagnostic study	Mixed/Not clearly specified	tNGS	Nanopore-based targeted sequencing test for direct tuberculosis identification, genot...; Overall assay validity 98% (102/104); key DR mutation agreement 100% vs Deeplex; TAT approximately 5–6 h
14	Sun et al., 2023	Observational diagnostic study	BALF	tNGS	A preliminary evaluation of targeted nanopore sequencing technology for the detection...; tNGS sensitivity 89.6% (95% CI 77.3–96.5); higher than smear, culture, and Xpert
15	Yu et al., 2024	Observational diagnostic study	Extrapulmonary specimens	tNGS	Targeted nanopore sequencing using clinical specimens for rapid diagnosis of extrapul...; EPTB Se/Sp/PPV/NPV/AUC = 86.4%/87.5%/97.3%/55.3%/0.87 (N=149)

ID	Author, year	Design	Specimen	Seq	Main finding / endpoint note
16	Gao et al., 2024	Observational diagnostic study	Tissue	tNGS	Nanopore-based targeted next-generation sequencing of tissue samples for tuberculosis...; Sensitivity 88.2%, specificity 94.1%, AUC 0.91, PPV 100% (12/12)
17	Zhou et al., 2024	Retrospective observational study	BALF	tNGS	Determination of the diagnostic accuracy of nanopore sequencing using bronchoalveolar...; Se/Sp/PPV/NPV/AUC = 90.70%/84.85%/92.13%/82.35%/0.88; AUC higher than smear, culture, Xpert, and CapitalBio
18	Cheng et al., 2025	Observational diagnostic study	Respiratory specimens	tNGS	Nanopore-targeted sequencing: A new and effective technique for the diagnosis of non-...; NTM-PD tNGS sensitivity 88.2% (vs culture 74.0% and smear 37.8%); AUC 0.893
19	Lin et al., 2024	Observational diagnostic study	Respiratory specimens	Nanopore sequencing (unspecified)	Diagnostic value of nanopore sequencing technology in nontuberculous mycobacterial pu...; NTM-PD Se/Sp/PPV/NPV/AUC = 88.9%/87.5%/77.4%/94.2%/0.882
20	Ou et al., 2025	Prospective observational study	Respiratory specimens	Nanopore sequencing (unspecified)	Study on the early diagnostic value of nanopore sequencing in alveolar lavage fluid s...; N=103; positivity (nanopore/culture/TB-DNA) = 73.8%/13.6%/33.0%
21	Ye et al., 2024	Observational diagnostic study	BALF	tNGS	Clinical application of nanopore-targeted sequencing technology in bronchoalveolar la...; N=223; Se (tNGS/CMT) = 74.83%/33.11% (P<0.001)
22	Chen et al., 2025	Observational diagnostic study	Mixed/Not clearly specified	tNGS	Diagnosis of drug-resistant tuberculosis: Rapid evaluation of drug susceptibility wit...; DR-TB (15 drugs): agreement (isolates/direct) = 94.3%/98.0%; kappa = 0.847/0.904
23	Fan et al., 2026	Prospective observational study	Respiratory specimens	tNGS	Diagnostic performance of nanopore-targeted sequencing for pulmonary infections in a ...; N=283 (72 polymicrobial); Se/Sp (MTB/NTM/fungi/bacteria) = 83.0/99.4, 89.8/98.2, 92.9/91.1, 97.4/57.8%; polymicrobial complete detection 77.8% vs 62.5%
24	Yang et al., 2025	Guideline/policy document	Mixed/Not clearly specified	Policy/guidance context	Nanopore-based targeted sequencing (NTS) for drug-resistant tuberculosis: an integrat...; Guidance/review context (source title uses NTS; no primary diagnostic accuracy cohort)
25	Schwab et al., 2024	Systematic review/meta-analysis	Mixed/Not clearly specified	tNGS	Targeted next-generation sequencing to diagnose drug-resistant tuberculosis: a system...; [Not extractable] Pooled Se/Sp endpoint stated; numerical values unavailable from currently accessible source
26	Li et al., 2023	Systematic review/meta-analysis	Mixed/Not clearly specified	mNGS	Metagenomic next-generation sequencing for Mycobacterium tuberculosis complex detecti...; [Not extractable] Pooled Se/Sp endpoint stated; numerical values unavailable from currently accessible source
27	Liu et al., 2023	Observational diagnostic study	Mixed/Not clearly specified	mNGS	Clinical application of metagenomic next-generation sequencing in tuberculosis diagnosis; mNGS detection 75% (39/52); clinical Se/Sp/PPV/NPV = 100%/61.9%/79.5%/100%
28	Sun et al., 2021	Observational diagnostic study	Extrapulmonary specimens	mNGS	Clinical efficacy of metagenomic next-generation sequencing for rapid detection of My...; mNGS Se/Sp/PPV/NPV/AUC = 56.11%/100%/100%/26.17%/0.79
29	Liu et al., 2021	Observational diagnostic study	BALF	mNGS	Tuberculosis diagnosis by metagenomic next-generation sequencing on bronchoalveolar l...; Untreated subgroup (n=253): mNGS sensitivity 59.9%, specificity 100.0%
30	You et al., 2024	Systematic review/meta-analysis	Respiratory specimens	mNGS	Diagnostic accuracy of metagenomic next-generation sequencing in pulmonary tuberculos...; [Not extractable] Accuracy endpoint stated; numerical values unavailable from currently accessible source
31	Gao et al., 2024	Observational diagnostic study	BALF	mNGS	The value of metagenomic next-generation sequencing for the diagnosis of pulmonary tu...; Se/Sp/PPV/NPV/AUC = 78.95%/100%/100%/94.87%/0.900; culture+mNGS AUC 0.933

ID	Author, year	Design	Specimen	Seq	Main finding / endpoint note
32	{World Health Organization} et al., 2023	Guideline/policy document	Mixed/Not clearly specified	Policy/guidance context	Catalogue of mutations in Mycobacterium tuberculosis complex and their association wi...; [Not applicable] Guideline document; no primary-cohort diagnostic accuracy dataset
33	{World Health Organization} et al., 2024	Guideline/policy document	Mixed/Not clearly specified	Policy/guidance context	WHO operational handbook on tuberculosis: module 3: diagnosis: rapid diagnostics for ...; [Not applicable] Operational handbook; no primary-cohort diagnostic accuracy dataset
34	{World Health Organization} et al., 2024	Organizational update/web notice	Mixed/Not clearly specified	Context-only source	WHO launches new guidance on the use of targeted next-generation sequencing tests for...; [Not applicable] Organizational update/news item; no primary-cohort diagnostic accuracy dataset
35	{World Health Organization} et al., 2021	Guideline/policy document	Mixed/Not clearly specified	Policy/guidance context	Catalogue of mutations in Mycobacterium tuberculosis complex and their association wi...; [Not applicable] Guideline catalogue; no primary-cohort diagnostic accuracy dataset
