# Supplementary material for: Nanopore Sequencing in Mycobacterial Diagnostics: Clinical and Laboratory Roles of mNGS and tNGS
Source: Diagnostics (Basel). 2026 Jun 15;16(12):1850. doi: 10.3390/diagnostics16121850 (PMC13297816; doi:10.3390/diagnostics16121850)
Supplement: Supplementary file 1 [file diagnostics-16-01850-s001.zip › diagnostics-4290692-supplementary/Table_S2_QUADAS2_Summary.pdf]

Table S2: QUADAS-2 domain-level judgments for original clinical studies (L=low risk/concern, H=high risk/concern, U=unclear). Signaling logic was tailored to direct-from-specimen sequencing and resistance-concordance contexts. “U” was assigned conservatively when reporting was insufficient to verify prespecified signaling criteria (e.g., sampling frame, blinding/threshold prespecification, interval handling, or participant flow completeness).

ID	Study	Patient selection	Index test	Reference standard	Flow/timing	Applicability
1	Schwab et al., 2025	U	U	U	U	U
3	Zhao et al., 2025	H	U	H	U	U
5	Murphy et al., 2023	U	U	U	U	U
6	Yang et al., 2026	L	U	U	U	L
8	Gui et al., 2026	H	U	H	U	U
9	Yu et al., 2025	H	U	U	U	U
10	Yan et al., 2024	H	U	U	U	U
11	Ren et al., 2024	H	U	H	U	U
12	Hall et al., 2023	U	U	U	U	U
13	Cabibbe et al., 2024	H	U	U	U	U
14	Sun et al., 2023	H	U	H	U	U
15	Yu et al., 2024	H	U	H	U	U
16	Gao et al., 2024	H	U	H	U	U
17	Zhou et al., 2024	H	U	H	U	U
18	Cheng et al., 2025	U	U	U	U	U
19	Lin et al., 2024	H	U	H	U	U
20	Ou et al., 2025	H	U	H	U	U
21	Ye et al., 2024	H	U	U	U	U
22	Chen et al., 2025	U	U	U	U	U
23	Fan et al., 2026	H	U	U	U	U
27	Liu et al., 2023	H	U	H	U	U
28	Sun et al., 2021	H	U	H	U	U
29	Liu et al., 2021	H	U	H	U	U
31	Gao et al., 2024	H	U	U	U	U
